# Risk stratification and determinant identification of high-need, high-cost ICU patients using machine learning: a large-scale retrospective study from a multi-specialty ICU in a tertiary hospital

**DOI:** 10.3389/fpubh.2026.1783334

**Published:** 2026-05-29

**Authors:** Yufei Hou, Qichao Shi, Cheng Wang

**Affiliations:** Department of Health Economics, General Hospital of Northern Theater Command, Shenyang, China

**Keywords:** health care costs, high-cost patients, intensive care units, machine learning, resource utilization

## Abstract

**Background:**

Intensive care units (ICU) account for a disproportionate share of hospital costs. Retrospectively identifying high-need, high-cost (HNHC) ICU patients using machine learning (ML) may inform structured cost auditing and more efficient healthcare resource allocation.

**Methods:**

This retrospective study included adult patients with ICU admission (≥24 h) from multiple specialty ICUs in a Chinese tertiary hospital (2018–2024). HNHC patients were defined as the top 5% of annual ICU costs. Clinical, laboratory, and resource-use variables were extracted and preprocessed. Six ML models were developed using feature selection, class balancing, cross-validation, and Bayesian optimization, with performance evaluated by area under the receiver operating characteristic curve (AUC) and related metrics. Shapley Additive Explanations (SHAP) was applied for model interpretability.

**Results:**

Among 51,056 ICU patients, 2,556 (5.0%) were classified as HNHC. HNHC patients had longer ICU stays, greater disease complexity, and substantially higher use and duration of intensive therapies, resulting in markedly increased total and ICU-related costs. After correlation and Boruta feature selection, 19 variables were retained for model development. Among six ML models, the random forest (RF) achieved the highest discriminative performance in the independent test set, with an AUC of 0.942 (95% CI 0.931–0.952), followed by Extra Trees and LightGBM. The random forest model showed a favorable balance between sensitivity and F1 score in this highly imbalanced population. Decision curve analysis demonstrated stable net benefit across clinically relevant threshold probabilities. SHAP interpretation identified ICU length of stay and mechanical ventilation duration as the strongest contributors, revealing pronounced nonlinear effects, while diagnosis-related group (DRG) reform did not substantially alter the contribution patterns of key features. Stratified analyses confirmed that model performance and feature contribution patterns remained stable across the pre- and post-DRG reform periods.

**Conclusion:**

High ICU costs were primarily associated with intensive resource use rather than demographics alone. A RF model reliably classified HNHC patients and remained stable across DRG reform, supporting its use as a tool for retrospective risk stratification, identification of cost drivers, and more efficient allocation of critical care resources.

## 1 Introduction

The intensive care unit (ICU) is among the most resource-intensive and clinically critical components of hospital care, providing treatment for critically ill patients who require continuous and comprehensive monitoring of their physiological status and health parameters (1). Although ICU services account for a relatively small proportion of overall healthcare utilization, they contribute disproportionately to total inpatient healthcare expenditures (2). Accumulating evidence indicates that healthcare costs are highly concentrated among a small subset of patients, commonly referred to as high-need, high-cost (HNHC) patients, who are typically defined as individuals in the top 5% of annual healthcare spending (3). Against the backdrop of rapid population aging and the growing number of HNHC patients admitted to ICUs, there is an urgent need to develop effective machine-learning (ML)–based approaches to retrospectively characterize HNHC ICU patients and to inform structured resource management and cost-driver identification (4).

Previous studies have shown that analyses of healthcare costs among HNHC patients are influenced by multiple factors, including heterogeneity in study design, case-mix composition, cost estimation methods, and the predictive performance of the models employed (2, 5). In recent years, ML techniques have demonstrated substantial advantages in medical prediction tasks (6). Compared with traditional statistical approaches, ML models are capable of handling high-dimensional data, capturing complex nonlinear relationships, and achieving superior predictive accuracy (7). Clinical characteristics and outcomes often exhibit nonlinear associations, which can be more effectively modeled using ML algorithms (8, 9). Currently, a variety of ML methods—including random forests (RF), gradient boosting machines, and XGBoost—have been widely applied to disease prognosis evaluation, in-hospital mortality prediction, and analyses of healthcare resource utilization (10). Most prior studies have developed models for HNHC patients using health insurance claims or general inpatient data; however, those predictive models specifically targeting HNHC patients in the ICU setting remain scarce. Moreover, there is a notable lack of studies that systematically compare the performance of multiple ML models using large-scale real-world data (11, 12).

ML models are often regarded as “black boxes” due to their limited interpretability, which has constrained their adoption in clinical management and decision-support systems (13). Explainable ML approaches, such as Shapley Additive Explanations (SHAP), enable the automatic identification of latent feature relationships in high-dimensional data and quantify the contribution of individual variables to model predictions (14). Through SHAP analysis, key predictors of HNHC ICU patient status—such as age and length of hospital stay—can be ranked according to their relative importance, and their specific contributions to the probability of being classified as an HNHC patient can be quantitatively assessed (3).

Beyond healthcare-specific interpretability, a broader stream of machine learning research has emphasized the importance of distinguishing stable, task-relevant determinants from incidental or context-dependent variation. For instance, recent work on content-independent handwriting authentication has shown that robust identity recognition can be achieved by abstracting stable handwriting style from variable written content (15). Related work on shift-stable prediction in healthcare AI (16) and on domain generalization more broadly (17) has similarly emphasized the need to identify predictive signals that remain stable under distributional change. In ICU cost prediction, the implementation of payment reforms—such as diagnosis-related group (DRG)-based reimbursement—constitutes an analogous contextual shift: while coding practices and accounting structures may evolve, the underlying clinical determinants of high resource use—organ-support intensity, treatment duration, and disease complexity—are expected to remain biologically and operationally stable. Examining whether SHAP-derived feature contributions and overall model performance persist across such reforms therefore offers a meaningful test of whether the identified cost drivers are genuinely intrinsic to critical care rather than artifacts of a particular reimbursement context.

Building on this perspective and using retrospective data from 51,056 ICU admissions across multiple specialty units of a tertiary hospital in China between 2018 and 2024, this study developed and evaluated interpretable ML models for retrospectively classifying HNHC ICU patients. We systematically compared multiple algorithms in this large real-world cohort, applied SHAP to characterize the nonlinear contribution patterns of clinical and resource-use determinants, and assessed whether model performance and feature attributions remained stable across the implementation of DRG–based payment reform—an aspect rarely examined in existing HNHC prediction studies. Our findings provide hospital administrators with practical, ICU-focused evidence to support data-driven decision-making in resource allocation and cost management.

## 2 Methods

### 2.1 Data source

This retrospective study initially identified 55,289 ICU admission records from adult patients admitted to multiple specialty ICUs at a tertiary hospital in China between January 1, 2018, and December 31, 2024. The participating units included the emergency ICU, neurological ICU, neurosurgical ICU, cardiovascular medical/surgical ICUs, and respiratory ICU.

We applied strict inclusion and exclusion criteria to ensure data quality. First, patients aged <18 years (*n* = 1,146) and those with an ICU length of stay (LOS) < 24 h (*n* = 41) were excluded, leaving 54,102 eligible cases. Subsequently, we addressed missing data and outliers through a rigorous preprocessing pipeline:

Missing values were identified in four laboratory parameters measured within the first 24 h of ICU admission: serum creatinine, platelet count, C-reactive protein (CRP), and absolute lymphocyte count. CRP exhibited the highest missing rate (~15%), while the others ranged from 3 to 5%, all well below our predefined exclusion threshold of 30%. To address these gaps while preserving sample size, we employed Multiple Imputation by Chained Equations (MICE) using the IterativeImputer from Python’s scikit-learn library. This method iteratively models each feature with missing values as a function of other features (including demographics and clinical variables) over 10 iterations with a fixed random seed for reproducibility. Because the imputed variables were four standard clinical laboratory markers with well-defined reference ranges, were used as predictors rather than the outcome variable, and exhibited limited missingness, imputation was performed on the full eligible cohort prior to train-test splitting to maximize information utilization and imputation accuracy. We acknowledge that fitting the imputation model exclusively within the training set would represent the methodological best practice for completely eliminating any potential information leakage. To empirically assess whether our design choice materially affected model generalization, we evaluated the optimal model on a temporally independent post-DRG-reform subgroup (Section 2.5)—a test set fully separated in time from the pre-reform training period—which serves as a stringent out-of-sample evaluation; corresponding results are reported in Section 3.4. All other variables, including demographics, treatment-related factors, costs, and outcomes, were complete without missing data.

Following imputation, we further excluded records with extreme, biologically implausible laboratory values (*n* = 1,933) identified using the 3-sigma rule (3 × IQR) combined with expert clinical judgment. Finally, patients with extreme cost outliers (*n* = 1,113), defined as records with zero cost or values clearly inconsistent with clinical reality, were removed. A total of 51,056 patients were ultimately included in the final analysis (Supplementary Figure S1).

### 2.2 Variable definition

The primary outcome of this study was HNHC status based on ICU-specific costs, defined as the direct medical expenses incurred strictly during the ICU stay, excluding general ward or other hospitalization costs. HNHC patients were identified as those whose ICU-specific costs ranked in the top 5% within each admission calendar year. Specifically, we calculated the 95th percentile of ICU costs for each year from 2018 to 2024 to serve as the annual HNHC threshold. These thresholds ranged from 138,345 to 158,549 Chinese Yuan (CNY) (detailed annual thresholds are provided in Supplementary Table S1). We adopted annual-specific thresholds rather than a single unified threshold across the entire study period to eliminate the potential confounding effects of temporal changes in cost structures, inflation, and healthcare policy adjustments (such as the DRG payment reform). This approach ensured a consistent classification proportion (~5%) of HNHC patients for each year. For patients whose ICU stay spanned across calendar years (*n* = 425, accounting for 0.83% of the total cohort), their total ICU costs were attributed to the calendar year of ICU admission.

Based on clinical expertise and prior literature, a comprehensive set of candidate predictors was extracted from electronic medical records and cost-settlement databases to capture the multidimensional factors influencing ICU-related healthcare costs. These variables were categorized as follows:

*Patient demographics and administrative characteristics*: Age, sex, payment method, source of ICU admission, ICU type, and DRG implementation status (with January 1, 2021, as the DRG policy implementation date).

*Proxies for disease severity*: Because comprehensive critical illness severity scores such as the Sequential Organ Failure Assessment (SOFA) score or Acute Physiology and Chronic Health Evaluation II (APACHE II) score were unavailable in the dataset, alternative indicators reflecting organ dysfunction and illness severity were selected based on prior evidence (18). These included primary diagnosis, number of secondary diagnoses, postoperative ICU admission status, and laboratory parameters—serum creatinine, platelet count, CRP, and absolute lymphocyte count. All laboratory values were defined as the first available measurement recorded within 24 h of ICU admission. For patients admitted via the emergency department, the first recorded value may have been obtained during the initial emergency evaluation prior to ICU transfer. When multiple measurements were available within this window, only the first chronological value was used; worst or peak values were not selected.

Indicators of treatment intensity and resource utilization: including endotracheal intubation, use of continuous renal replacement therapy (CRRT), extracorporeal membrane oxygenation (ECMO), blood transfusion, mechanical ventilation (MV), ICU LOS, hours of MV, CRRT hours, and ECMO hours.

### 2.3 Data processing and feature selection

To ensure the validity and robustness of model development, all predictive variables underwent systematic preprocessing and feature selection within a rigorous multi-stage analytical pipeline. Crucially, the dataset was first randomly split into a training set (80%) and a testing set (20%). All subsequent preprocessing steps—including standardization, encoding, and feature selection—were strictly performed within the training set only to prevent data leakage. The testing set was kept completely isolated and was not involved in any fitting or selection processes.

For continuous variables, standardization was applied by subtracting the mean and dividing by the standard deviation (*Z*-score normalization). The scaling parameters (mean and standard deviation) were fitted exclusively on the training set and then applied to transform the testing set. This procedure enhanced comparability across features with different measurement scales and mitigated the influence of scale heterogeneity on model training. Categorical variables were encoded using one-hot encoding to generate dummy variables.

To reduce feature redundancy and the risk of overfitting, Spearman correlation analysis was first conducted within the training set to assess collinearity among variables. When the correlation coefficient between two variables exceeded 0.9, the feature more strongly associated with the outcome was retained. Subsequently, the Boruta algorithm was applied on the training set for feature selection, with the optimal feature subset determined via 10-fold cross-validation.

In each iteration, the Boruta algorithm evaluates the importance of each feature relative to randomized “shadow features.” Features with importance significantly higher than the maximum importance of shadow features (ShadowMax) are confirmed as important; those significantly lower than the minimum shadow importance (ShadowMin) are rejected; and those falling between ShadowMin and ShadowMax are classified as tentative and subject to further evaluation (19). Feature selection was implemented using the Boruta package in R, with parameters set to maxRuns = 100 and ntree = 1,000.

### 2.4 Development and evaluation of ML models

In this study, six ML models representing distinct computational paradigms were constructed and compared to ensure robust identification of HNHC patients. The selected algorithms included Logistic Regression (LR), RF, Extra Trees (ET), *K*-Nearest Neighbors (KNN), XGBoost, and LightGBM. LR was employed as a linear baseline due to its high interpretability, whereas KNN was utilized to capture local non-linear patterns without assuming an underlying data distribution (20). To address complex non-linear relationships and interactions, we incorporated ensemble methods: RF and ET (bagging-based algorithms) were chosen to reduce variance by aggregating predictions from multiple decorrelated decision trees; conversely, XGBoost and LightGBM (gradient boosting-based algorithms) were selected to enhance predictive performance by sequentially minimizing a regularized loss function (21, 22). The detailed algorithmic principles, rationale for selection, hyperparameter search ranges, and final optimized values for each model are provided in Supplementary Table S2. All models were implemented in a Python 3.9.13 environment using the scikit-learn library and relevant boosting frameworks.

To address the significant class imbalance between the HNHC group (~5%) and the non-HNHC group, we applied the Synthetic Minority Over-sampling Technique (SMOTE) to the training data. Specifically, SMOTE generates synthetic samples for the minority class (HNHC) by linearly interpolating between existing samples and their *k*-nearest neighbors (*k* = 5) in this study, thereby balancing the training set distribution to a 1:1 ratio and preventing classifier bias toward the majority class (non-HNHC). Crucially, SMOTE was strictly applied only within the training set, while the testing set remained untouched, to prevent data leakage. We selected SMOTE over other techniques for four primary reasons: (1) unlike random oversampling, SMOTE creates synthetic rather than duplicated samples, reducing the risk of overfitting; (2) unlike undersampling, it preserves all information from the majority class, which is vital for our large-scale dataset (>50,000 cases); (3) compared to cost-sensitive learning, SMOTE avoids the need for a predefined misclassification cost matrix, which is challenging to quantify precisely; and (4) as a model-agnostic preprocessing step, it allows for a fair and consistent comparison across all six candidate algorithms (23, 24). To further validate this choice, we conducted a sensitivity analysis comparing six class imbalance handling strategies: no resampling, random oversampling, SMOTE, Adaptive Synthetic Sampling (ADASYN), cost-sensitive learning (class weight adjustment), and SMOTE combined with Tomek links (SMOTE + Tomek). All strategies were evaluated using the RF model with identical optimized hyperparameters, and classification thresholds were determined on the training set to ensure a fair comparison (detailed in Results Section 3.5).

Hyperparameters for all models were fine-tuned using Bayesian Optimization with 5-fold cross-validation, targeting the F1-score as the objective function over 50 iterations per model. The classification threshold was not fixed at the default 0.5; instead, we employed an F1-score optimization strategy, identifying the optimal cutoff by grid-searching candidate thresholds on the training set. This approach was chosen because the F1-score, as the harmonic mean of precision and recall, better balances the trade-off between false positives and false negatives in low-prevalence scenarios, avoiding the potential bias of traditional metrics. Taking the optimal RF model as an example, the final tuned parameters included: n_estimators = 177 (search range: 100–300), max_depth = 20 (range: 5–20), min_samples_split = 6 (range: 5–30), min_samples_leaf = 2 (range: 2–15), and max_features = ‘sqrt’. Mechanistically, min_samples_split and min_samples_leaf were tuned to restrict tree complexity and prevent overfitting, while max_features = ‘sqrt’ reduced inter-tree correlation by limiting the feature subset at each split. Full hyperparameter settings for all models are detailed in Supplementary Table S2.

Model performance was comprehensively evaluated on the independent testing set using metrics derived from the confusion matrix, which comprises four fundamental elements: True Positives (TP), True Negatives (TN), False Positives (FP), and False Negatives (FN). The specific evaluation metrics included: Sensitivity $\frac{\mathrm{TP}}{\mathrm{TP}+\mathrm{FN}}$ , Specificity $\frac{\mathrm{TN}}{\mathrm{TN}+\mathrm{FP}}$ , Precision $\frac{\mathrm{TP}}{\mathrm{TP}+\mathrm{FP}}$ , Accuracy $\frac{\mathrm{TP}+\mathrm{TN}}{\mathrm{TP}+\mathrm{TN}+\mathrm{FP}+\mathrm{FN}}$ , and the F1-score $\frac{2\times \text{Precision}\times \text{Recall}}{\text{Precision}+\text{Recall}}$ (25). Given the low prevalence of HNHC outcome events (~5%), we additionally reported Average Precision (AP), to provide a more robust assessment of the models’ ability to identify the minority class in imbalanced datasets. Furthermore, to evaluate the reliability of predicted probabilities, we utilized Calibration Plots and the Brier Score (range: 0–1, with lower scores indicating better calibration) to quantify the agreement between predicted risks and observed outcomes. Uncertainty in performance estimates was assessed using 95% confidence intervals (CIs) derived from 1,000 bootstrap resamples.

### 2.5 Interpretability of ML

To elucidate the prediction mechanism of the best-performing model (RF) and enhance clinical interpretability, the SHAP method was applied to quantify the marginal contribution of each input variable to the predicted probability of HNHC. To assess the relative importance of predictors at the population level, the mean absolute SHAP value for each variable was calculated in the test set and used as a quantitative measure of global feature importance. In addition, SHAP summary plots were generated to illustrate the distribution of feature effects on model outputs across different value ranges.

Given the implementation of the DRG payment reform at our institution on January 1, 2021, which could potentially alter cost accounting practices and model output patterns, we rigorously assessed the stability of the optimal RF model across the pre- and post-reform periods. The testing set was stratified into two subgroups based on the DRG implementation status: a pre-reform subgroup (2018–2020, DRG = 0) and a post-reform subgroup (2021–2024, DRG = 1). Model performance within each subgroup was evaluated using AUC, AP, Brier Score, Sensitivity, Specificity, and F1-Score. A formal statistical comparison of the AUC difference between the two periods was conducted by constructing the 95% CI of the difference via bootstrapping; if the interval included zero, the difference was considered statistically non-significant. Additionally, stratified ROC curves and Calibration Plots were generated to visualize performance discrepancies.

To further investigate whether the DRG reform altered the contribution patterns of key predictors, we calculated SHAP values for all 19 features using the full testing set. The distributions of SHAP values were then grouped by the pre- and post-reform periods and compared using the Mann–Whitney *U* test. We also assessed the magnitude of practical differences by comparing the mean absolute SHAP values between the two groups. It is important to note that DRG reform might affect not only predictive variables but also the cost accounting system itself, potentially redefining the outcome. To mitigate this, our study defined HNHC status based on a relative annual ranking (top 5% of costs within each year) rather than a fixed absolute cost threshold. This relative definition inherently adjusts for systemic shifts in cost accounting over time, ensuring a consistent and comparable outcome classification across the study period.

### 2.6 Statistical analysis

Categorical variables are summarized as frequencies and percentages. Normality testing indicated that all continuous variables were non-normally distributed and are therefore presented as medians with interquartile ranges [M (IQR)]; between-group comparisons were performed using the Mann–Whitney *U* test. Comparisons of categorical variables were conducted using the chi-square test or Fisher’s exact test, as appropriate. All statistical tests were two-sided, and a *p-*value < 0.05 was considered statistically significant.

Data cleaning, feature selection, and the development and evaluation of ML models were performed using Python 3.9.13, with the aid of libraries including pandas, scikit-learn, and XGBoost. Model interpretability analyses were conducted using the SHAP library. Statistical analyses and data visualization were carried out using a combination of Python 3.9.13 and R version 4.5.1.

## 3 Results

### 3.1 Descriptive analysis

A total of 51,056 ICU patients were included in this study, of whom 48,500 (95.0%) were classified as non-HNHC and 2,556 (5.0%) as HNHC. No statistically significant difference was observed in sex distribution between the two groups (*p* = 0.124). Patients in the HNHC group were slightly older than those in the non-HNHC group [62.0 (52.0–69.0) vs. 61.0 (53.0–68.0) years, *p* = 0.005]. Health insurance type differed significantly between groups (*p* < 0.001), whereas the proportion of DRG-based payment was identical in the non-HNHC and HNHC groups (both 61.6%, *p* = 1.000) (Table 1).

**Table 1:** Baseline predictive variables for high-need, high-cost (HNHC) ICU patients.

Variables	Overall (*N* = 51,056)	non-HNHC (*n* = 48,500)	HNHC (*n* = 2,556)	*p*-value
Sample size, *n*	51,056	48,500	2,556	
Demographic characteristics				
Sex, *n* (%)				0.124
Male	34,003 (66.6)	32,337 (66.7)	1,666 (65.2)	
Female	17,053 (33.4)	16,163 (33.3)	890 (34.8)	
Age, median (IQR)	61.0 (53.0–68.0)	61.0 (53.0–68.0)	62.0 (52.0–69.0)	0.005
Health insurance				
Type of medical insurance, *n* (%)				<0.001
Urban employee medical insurance	45,929 (90.0)	43,684 (90.1)	2,245 (87.8)	
Urban–rural resident medical insurance	363 (0.7)	345 (0.7)	18 (0.7)	
Self-pay	3,447 (6.8)	3,202 (6.6)	245 (9.6)	
Other	1,317 (2.6)	1,269 (2.6)	48 (1.9)	
DRG, *n* (%)				1.000
No	19,601 (38.4)	18,620 (38.4)	981 (38.4)	
Yes	31,455 (61.6)	29,880 (61.6)	1,575 (61.6)	
ICU-related characteristics				
Source of ICU admission, *n* (%)				<0.001
Outpatient clinic	18,355 (36.0)	17,671 (36.4)	684 (26.8)	
Emergency department	32,701 (64.0)	30,829 (63.6)	1872 (73.2)	
ICU type, *n* (%)				<0.001
Respiratory ICU	2,515 (4.9)	2,418 (5.0)	97 (3.8)	
Emergency ICU	3,092 (6.1)	2,653 (5.5)	439 (17.2)	
Neurology ICU	6,257 (12.3)	6,108 (12.6)	149 (5.8)	
Neurosurgical ICU	11,164 (21.9)	10,687 (22.0)	477 (18.7)	
Cardiology ICU	11,454 (22.4)	11,105 (22.9)	349 (13.7)	
Cardiac surgery ICU	16,574 (32.5)	15,529 (32.0)	1,045 (40.9)	
ICU LOS, median (IQR)	2.0 (1.0–4.0)	2.0 (1.0–4.0)	8.0 (3.0–17.0)	<0.001
Postoperative ICU admission, *n* (%)				<0.001
No	19,128 (37.5)	17,666 (36.4)	1,462 (57.2)	
Yes	31,928 (62.5)	30,834 (63.6)	1,094 (42.8)	
Disease complexity				
Primary diagnosis, *n* (%)				<0.001
Injury, poisoning, external causes, or surgery	2,673 (5.2)	2,424 (5.0)	249 (9.7)	
Neoplasms and hematologic diseases	2,392 (4.7)	2,374 (4.9)	18 (0.7)	
Circulatory and respiratory system diseases	41,163 (80.6)	39,136 (80.7)	2027 (79.3)	
Digestive and endocrine system diseases	615 (1.2)	596 (1.2)	19 (0.7)	
Neurological and psychiatric diseases	1,129 (2.2)	1,046 (2.2)	83 (3.2)	
Infectious and communicable diseases	398 (0.8)	339 (0.7)	59 (2.3)	
Other	2,686 (5.3)	2,585 (5.3)	101 (4.0)	
Number of secondary diagnoses, median (IQR)	3.0 (2.0–5.0)	3.0 (2.0–5.0)	5.0 (2.0–7.0)	<0.001
Therapeutic interventions				
Endotracheal intubation, *n* (%)				<0.001
No	42,894 (84.0)	41,403 (85.4)	1,491 (58.3)	
Yes	8,162 (16.0)	7,097 (14.6)	1,065 (41.7)	
Mechanical ventilation, *n* (%)				<0.001
No	28,108 (55.1)	27,512 (56.7)	596 (23.3)	
Yes	22,948 (44.9)	20,988 (43.3)	1960 (76.7)	
Mechanical ventilation (hours), median (IQR)	20.9 (17.2–47.4)	20.7 (16.9–43.2)	131.1 (41.0–341.6)	<0.001
CRRT, *n* (%)				<0.001
No	49,719 (97.4)	47,524 (98.0)	2,195 (85.9)	
Yes	1,337 (2.6)	976 (2.0)	361 (14.1)	
CRRT (hours), median (IQR)	30.0 (12.5–63.5)	22.0 (10.0–47.5)	66.0 (31.0–116.0)	<0.001
ECMO, *n* (%)				<0.001
No	49,581 (97.1)	47,317 (97.6)	2,264 (88.6)	
Yes	1,475 (2.9)	1,183 (2.4)	292 (11.4)	
ECMO(hours), median (IQR)	91.6 (44.6–163.7)	77.2 (40.8–134.6)	220.9 (130.5–327.6)	<0.001
Blood transfusion, *n* (%)				<0.001
No	37,365 (73.2)	36,267 (74.8)	1,098 (43.0)	
Yes	13,691 (26.8)	12,233 (25.2)	1,458 (57.0)	
Laboratory parameters				
Serum creatinine (μmol/L), median (IQR)	71.8 (58.1–90.5)	71.6 (58.1–89.8)	76.9 (58.7–107.6)	<0.001
Platelet count (×10^9^/L), median (IQR)	192.9 (151.0–237.0)	193.0 (152.0–238.0)	173.0 (124.0–225.0)	<0.001
CRP (mg/L), median (IQR)	37.3 (10.3–74.3)	36.6 (10.0–73.0)	54.1 (18.0–100.2)	<0.001
Absolute lymphocyte count (×10^9^/L), median (IQR)	0.9 (0.6–1.3)	0.9 (0.6–1.3)	0.8 (0.6–1.2)	<0.001

HNHC, high-need, high-cost; ICU, intensive care unit; IQR, interquartile range; DRG, diagnosis-related group; LOS, length of stay; MV, mechanical ventilation; CRRT, continuous renal replacement therapy; ECMO, extracorporeal membrane oxygenation; CRP, C-reactive protein.

Compared with the non-HNHC group, patients in the HNHC group were more frequently admitted to the ICU via the emergency department, exhibited a different distribution of ICU types, and had a significantly longer ICU LOS [8.0 (3.0–17.0) vs. 2.0 (1.0–4.0) days; all *p* < 0.001]. In addition, the proportion of postoperative ICU admissions was lower in the HNHC group (*p* < 0.001). Significant differences were also observed between the non-HNHC and HNHC groups in terms of primary diagnosis categories and the number of secondary diagnoses (*p* < 0.001) (Table 1).

With respect to therapeutic interventions, the HNHC group had significantly higher rates and longer hours of endotracheal intubation, MV, CRRT, ECMO, and blood transfusion than the non-HNHC group (all *p* < 0.001). Regarding laboratory parameters, patients in the HNHC group had higher serum creatinine and CRP levels, but lower platelet counts and absolute lymphocyte counts (all *p* < 0.001) (Table 1).

**Figure 1:**
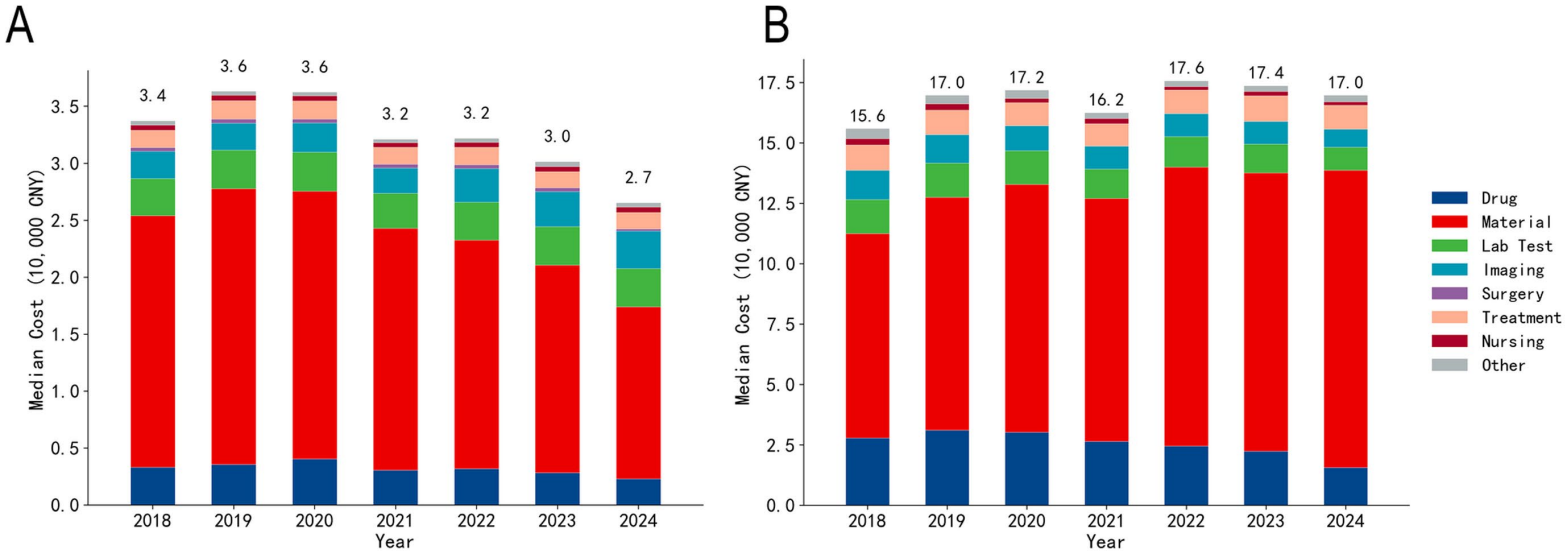
Temporal trends in median hospitalization cost components among ICU patients, 2018–2024. Median total hospitalization cost ranged from 2.7 to 3.6 (×10^4^ CNY) across the overall ICU cohort and from 15.6 to 17.6 (×10^4^ CNY) among HNHC patients. Material costs constituted the dominant component in both groups, with the disparity being most pronounced for HNHC patients. **(A)** Median hospitalization cost components for all ICU patients, by year. **(B)** Median hospitalization cost components for HNHC ICU patients, by year.

During the study period (2018–2024), the annual composition of hospitalization costs among ICU patients remained relatively stable overall (Figure 1A). Across the entire cohort, hospitalization costs were primarily driven by material costs, drug costs, and treatment-related expenses, with only modest year-to-year variation in the median total hospitalization cost. In contrast, hospitalization expenditures were substantially higher among HNHC patients, who also exhibited a distinct cost structure (Figure 1B). Within the HNHC group, material costs accounted for the largest proportion of total expenditures, followed by drug costs, treatment costs, and examination-related expenses; this cost composition pattern remained largely consistent across study years.

Further analyses demonstrated that the median total hospitalization cost (225,800 CNY) and ICU-specific cost (188,100 CNY) in the HNHC group were markedly higher than those in the non-HNHC group (73,000 and 40,400 CNY, respectively). Expenditures across all major cost components—including drug, material, laboratory testing, imaging, treatment, and nursing—were also significantly higher in the HNHC group (all *p* < 0.001). Detailed comparisons of individual cost components are provided in Supplementary Table S1.

### 3.2 Feature selection

**Figure 2:**
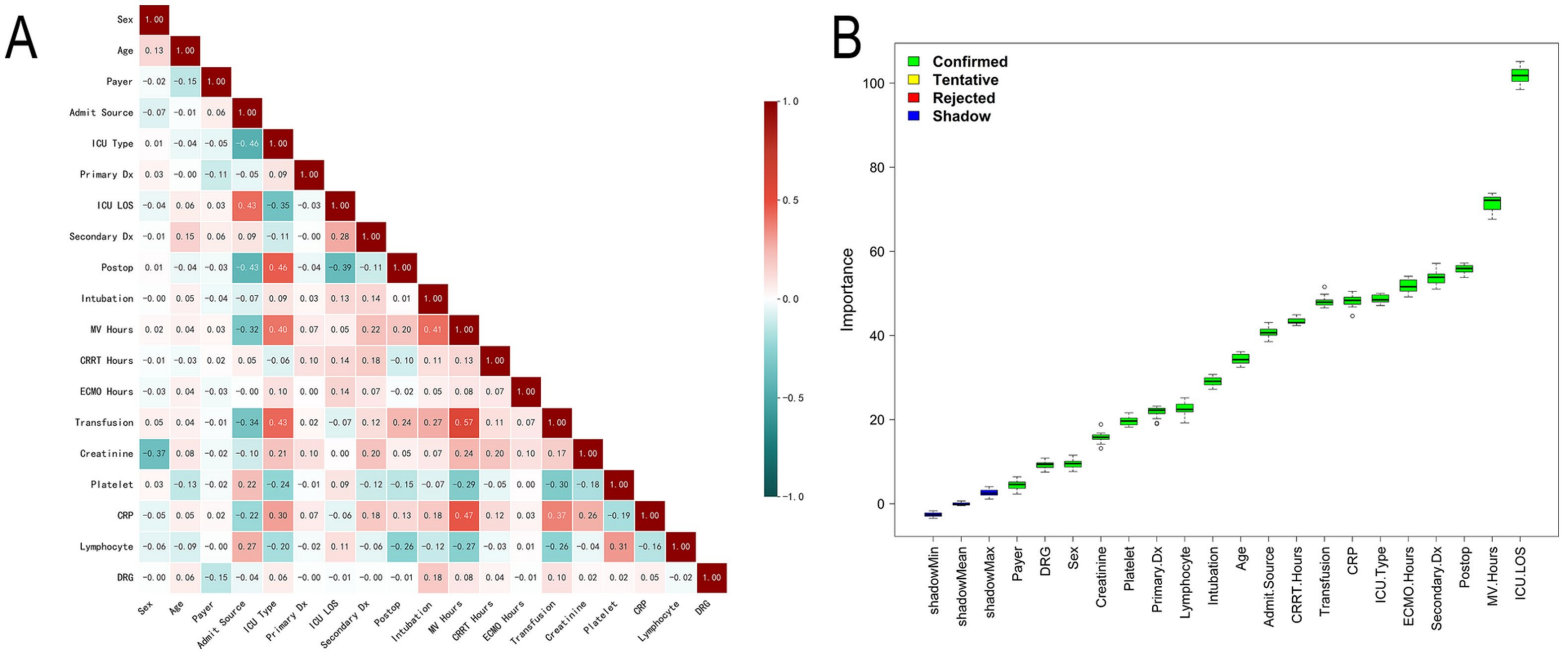
Feature selection and importance ranking of candidate variables. **(A)** Spearman correlation matrix among the 19 predictors retained after correlation-based filtering (which resolved three highly correlated variable pairs with |*ρ*| > 0.9). **(B)** Boruta feature importance distributions across iterations; all 19 features (green) were classified as confirmed important and retained for downstream model development, with ICU LOS and MV hours showing the highest importance.

The original feature set included 22 candidate independent variables: age, sex, payer, source of ICU admission, ICU type, DRG, primary diagnosis, number of secondary diagnoses, postoperative status, serum creatinine, platelet count, CRP, absolute lymphocyte count, intubation, MV status, MV hours, CRRT status, CRRT hours, ECMO status, ECMO hours, transfusion, and ICU LOS. Spearman correlation analysis identified three pairs of highly correlated predictors (|*ρ*| > 0.9). MV status was strongly correlated with MV hours (*ρ* = 0.944); because MV hours showed a stronger association with HNHC (0.220 vs. 0.146), the duration variable was retained and the binary indicator was excluded. Similar collinearity was observed between CRRT status and CRRT hours (*ρ* = 0.998) and between ECMO status and ECMO hours (*ρ* = 1.000); in both cases, the duration variables were retained due to their stronger associations with the outcome, and the corresponding binary variables (MV status, CRRT status, and ECMO status) were removed. After correlation-based filtering, 19 variables were retained for subsequent analyses (Figure 2A).

Based on this reduced feature set, further feature selection was performed using the Boruta algorithm. All 19 variables—sex, age, payer, source of ICU admission, ICU type, primary diagnosis, ICU LOS, secondary diagnoses, postoperative status, intubation, MV hours, CRRT hours, ECMO hours, transfusion, creatinine, platelet count, CRP, lymphocyte count, and DRG—were identified as important features and retained in the final model (Figure 2B).

### 3.3 Classification of HNHC ICU patients

**Figure 3:**
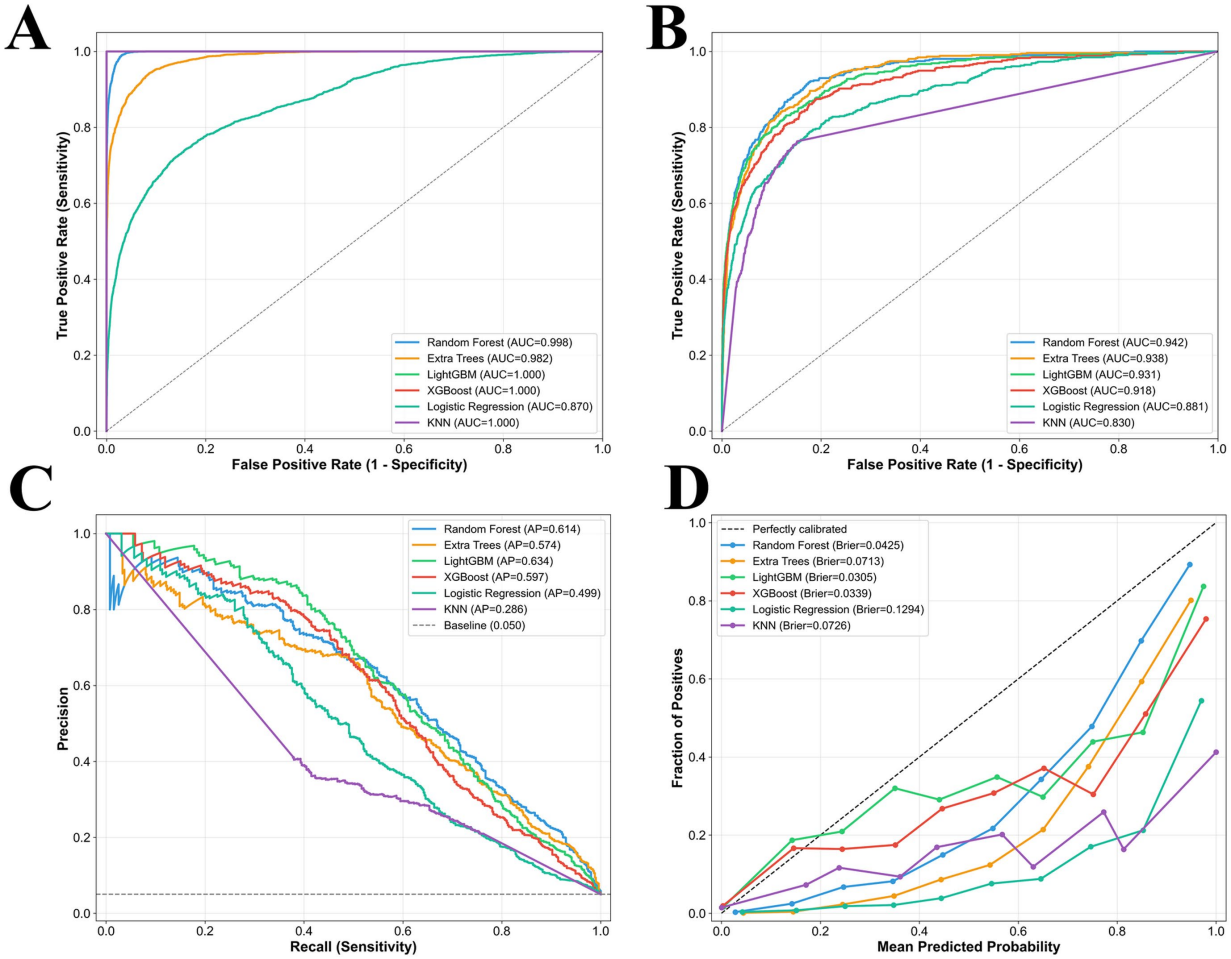
Performance comparison of six machine learning models. In the independent test set, random forest (RF) achieved the highest AUC (0.942, 95% CI 0.931–0.952), followed by Extra Trees (0.938) and LightGBM (0.931); Logistic Regression (0.881) and KNN (0.830) showed inferior discrimination. LightGBM achieved the highest average precision (AP = 0.634) and the lowest Brier score (0.0305), indicating the best calibration. **(A)** ROC curves in the training set; **(B)** ROC curves in the testing set; **(C)** precision-recall curves in the testing set; **(D)** Calibration plots in the testing set. AUC, area under the receiver operating characteristic curve; AP, average precision; KNN, *K*-nearest neighbors.

All six ML models were constructed using the same set of 19 input variables and evaluated in an independent test set. In the test cohort, HNHC patients accounted for approximately 5% of the population, indicating a highly imbalanced classification setting. The discriminative performance of the ML models in classifying HNHC ICU patients varied across algorithms (Figure 3 and Table 2). The RF model achieved the highest AUC of 0.942 (95% CI, 0.931–0.952). ET and LightGBM showed comparable performance, with AUCs of 0.938 (95% CI, 0.928–0.948) and 0.931 (95% CI, 0.919–0.942), respectively, whereas XGBoost achieved an AUC of 0.918 (95% CI, 0.904–0.931). In contrast, LR and KNN demonstrated inferior discriminative ability. In the training set, all models yielded high AUC values, indicating good overall model fit (Figure 3A). Given the class imbalance, AP was further examined (Figure 3C). LightGBM achieved the highest AP of 0.634, followed by RF (0.614), XGBoost (0.597), and ET (0.574), while LR (0.499) and KNN (0.286) performed substantially worse. Calibration assessment (Figure 3D) showed that LightGBM had the lowest Brier score (0.0305), followed by XGBoost (0.0339) and RF (0.0425), whereas LR exhibited the poorest calibration (Brier = 0.1294).

**Table 2:** Performance of machine learning models for predicting high-need, high-cost ICU patients in the test set.

Model	AUC	Sensitivity	Specificity	Precision	NPV	F1	MCC
Random forest	0.942 (0.931–0.952)	0.604 (0.558–0.646)	0.976 (0.973–0.979)	0.569 (0.528–0.610)	0.979 (0.976–0.982)	0.586 (0.550–0.620)	0.564 (0.527–0.599)
Extra Trees	0.938 (0.928–0.948)	0.521 (0.476–0.566)	0.984 (0.982–0.987)	0.637 (0.590–0.683)	0.975 (0.972–0.978)	0.573 (0.534–0.610)	0.556 (0.516–0.593)
LightGBM	0.931 (0.919–0.942)	0.625 (0.581–0.667)	0.971 (0.968–0.974)	0.532 (0.493–0.573)	0.980 (0.977–0.983)	0.575 (0.541–0.608)	0.552 (0.517–0.587)
XGBoost	0.918 (0.904–0.931)	0.648 (0.606–0.688)	0.958 (0.954–0.961)	0.447 (0.412–0.482)	0.981 (0.978–0.984)	0.529 (0.495–0.561)	0.509 (0.474–0.542)
Logistic regression	0.881 (0.863–0.898)	0.491 (0.447–0.534)	0.971 (0.968–0.975)	0.474 (0.432–0.517)	0.973 (0.970–0.977)	0.482 (0.443–0.519)	0.454 (0.414–0.492)
KNN	0.830 (0.809–0.851)	0.761 (0.722–0.800)	0.848 (0.841–0.855)	0.209 (0.192–0.228)	0.985 (0.983–0.988)	0.328 (0.305–0.353)	0.344 (0.321–0.369)

HNHC, high-need, high-cost; AUC, area under the receiver operating characteristic curve; NPV, negative predictive value; *F1*, *F1* score (harmonic mean of precision and recall); MCC, Matthews correlation coefficient; KNN, *K*-nearest neighbors.

With respect to classification performance (Table 2), an optimized threshold that maximized the F1 score was applied for each model. The RF model achieved a relatively balanced trade-off between sensitivity and specificity in the test set, with a sensitivity of 0.604 and an F1 score of 0.586. Although the ET and LightGBM models maintained high overall accuracy and specificity, their F1 scores were relatively lower (0.573 and 0.575, respectively), suggesting limited ability to identify a subset of HNHC patients. The XGBoost model showed higher sensitivity (0.648) but a lower F1 score (0.529) than the RF model, reflecting an increased proportion of false-positive predictions. In the context of the low prevalence of HNHC, both LR and KNN yielded F1 scores below 0.500, indicating overall limited classification performance.

Decision curve analysis is presented in Supplementary Figure S2. Given the low incidence of HNHC, clinically meaningful threshold probabilities were concentrated in the lower range. Across low to moderate threshold probabilities (approximately 0.05–0.30), multiple models demonstrated positive net benefit. LightGBM and XGBoost achieved relatively higher net benefit overall, whereas the RF model exhibited more stable net benefit across this range. As the threshold probability increased further (approximately >0.60), net benefit declined across all models and approached zero or became negative, indicating limited clinical utility of high-threshold strategies in low-event-rate settings.

Confusion matrix analyses revealed marked differences in error patterns among models (Supplementary Figure S3). The RF model maintained a relatively low false-positive rate while effectively identifying HNHC patients, whereas other models were characterized by insufficient sensitivity or increased false-positive rates, resulting in lower overall classification stability.

### 3.4 Model explanation based on SHAP

**Figure 4:**
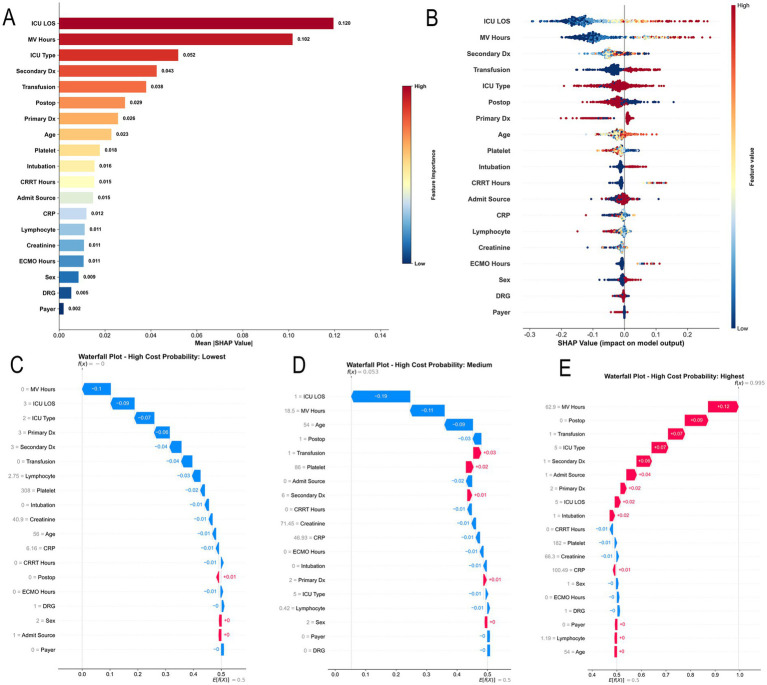
Model interpretability based on SHAP analysis. **(A)** Global feature importance ranked by mean absolute SHAP values. **(B)** SHAP summary plot showing the distribution of feature effects on model output, with feature values color-coded from low (blue) to high (red). **(C–E)** Individual SHAP waterfall plots for representative patients with the lowest **(C)**, medium **(D)**, and highest **(E)** predicted probabilities of HNHC status; feature values shown next to each row are on their original physical scales (units listed in Supplementary Figure S4 caption). SHAP, SHapley additive explanations; LOS, length of stay; MV, mechanical ventilation.

To further interpret model predictions and identify key contributing factors, SHAP was applied to explain the optimal model (Figures 4A,B). The global feature importance ranking indicated that ICU LOS (0.120) and MV hours (0.102) were the two strongest positive contributors to HNHC prediction, followed by ICU type, number of secondary diagnoses, transfusion status, and postoperative status. In contrast, demographic characteristics and several laboratory indices contributed relatively little to overall model output.

To illustrate the prediction logic at the individual level, representative patients with low, intermediate, and high predicted probabilities of HNHC were examined (Figures 4C–E). Patients with low predicted risk were primarily characterized by shorter ICU LOS, minimal or no MV hours, and absence of transfusion. For patients with intermediate predicted risk, positive and negative contributions tended to offset one another. In patients with high predicted risk, multiple positive factors accumulated—most notably prolonged MV hours, transfusion, specific ICU types, and relevant diagnostic features—resulting in a marked increase in predicted probability.

SHAP dependence plots demonstrated clear nonlinear positive relationships between ICU LOS and MV hours and model output, with age also exhibiting a nonlinear pattern; by contrast, most laboratory variables and demographic features showed weaker effects (Supplementary Figure S4). The stability of the RF model across the pre- and post-DRG reform periods was further assessed. The model maintained comparable performance in both periods: the pre-DRG subgroup achieved an AUC of 0.947 (95% CI, 0.932–0.961), AP of 0.624, and Brier score of 0.039, while the post-DRG subgroup achieved an AUC of 0.938 (95% CI, 0.923–0.950), AP of 0.612, and Brier score of 0.0448 (Supplementary Table S5 and Supplementary Figure S6). The bootstrapped AUC difference was not statistically significant (ΔAUC = 0.010; 95% CI, −0.011 to 0.030).

Mann–Whitney *U* tests comparing SHAP value distributions between the two periods (Supplementary Table S4) showed that, although 16 of 19 features reached statistical significance (*p* < 0.05), the absolute differences in mean SHAP values were negligible (all |Δmean SHAP| < 0.01), likely reflecting the large sample size rather than substantive changes in feature contributions. Overall, the direction and magnitude of feature effects remained stable across the reform periods (Supplementary Figure S5).

### 3.5 Sensitivity analysis of class imbalance handling strategies

To evaluate the robustness of the model to the choice of class imbalance handling strategy, six approaches were compared using the RF model with identical hyperparameters on the testing set (Supplementary Table S6). The six strategies—no resampling, random oversampling, SMOTE, ADASYN, cost-sensitive learning, and SMOTE+Tomek—yielded highly similar discriminative performance, with AUC values ranging from 0.941 to 0.945 and broadly overlapping 95% CIs. F1 scores ranged from 0.530 to 0.605, and AP values ranged from 0.608 to 0.672. These results indicate that the RF model’s classification performance was robust to the choice of resampling strategy, and the use of SMOTE did not introduce meaningful bias relative to alternative approaches.

## 4 Discussion

In a large multi-specialty ICU cohort of 51,056 admissions, we applied established ML and interpretability tools to address an ICU-specific question that remains underexplored in HNHC research: who drives disproportionate resource use within critical care, and does this pattern hold under payment reform. Although HNHC patients accounted for only 5.0% of the ICU population, they were characterized by older age, predominant emergency admissions, and a high dependence on organ support therapies—including CRRT, ECMO, and MV—leading to substantially increased healthcare expenditures, particularly material-related costs. In this highly imbalanced setting, the RF model outperformed LR and other ML algorithms, achieving an AUC of 0.942. SHAP-based analyses identified ICU LOS, MV hours, and selected physiological indicators as the primary contributors to high-cost utilization, and the model’s classification logic remained stable across the implementation of DRG-based payment reform—an aspect rarely examined in prior HNHC studies. The proportion of HNHC patients in our cohort (5.0%) is consistent with prior studies on the concentration of healthcare resource utilization, whereby a small subset of patients accounts for a disproportionate share of medical expenditures (26). Descriptive analyses revealed substantial differences in key predictors between the HNHC and non-HNHC groups, in line with previous reports (27, 28). Our findings indicate that HNHC patients not only exhibit more pronounced inflammatory responses—reflected by elevated CRP levels and reduced lymphocyte counts—and impaired coagulation function, as evidenced by thrombocytopenia, but are more notably characterized by a high dependence on invasive therapies. Compared with non-HNHC patients, the use and duration of mechanical ventilation, CRRT, and ECMO were markedly higher in the HNHC group. This divergence in treatment intensity was directly reflected in the cost structure: material costs constituted the largest proportion of total expenditures among HNHC patients, followed by medication costs. These findings suggest that high ICU costs are not associated solely by prolonged length of stay, but rather by the intensive use of high-cost medical consumables (e.g., CRRT and ECMO) and expensive pharmacological therapies. Consequently, retrospectively characterizing HNHC patients has implications not only for resource auditing and capacity planning, but also represents a critical leverage point for informing the use of high-value consumables and optimizing healthcare resource allocation.

Accurately classifying HNHC patients is challenging because of their low prevalence in the overall population (5%), which makes traditional statistical methods prone to overfitting the majority class (non-HNHC) while failing to adequately capture the minority class (HNHC). Many studies have relied on conventional approaches (29, 30), such as LR, to classify HNHC patients; however, their predictive performance has been shown to be inferior to that of ML-based models. In prior HNHC prediction studies, the best-performing methods and their corresponding discrimination—typically assessed using AUC—have included XGBoost (AUC, 0.801) (31), smoothed Bayesian network models (AUC, 0.840) (32), and LightGBM (AUC, 0.900) (33). Although the optimal model may vary across populations, datasets, and modeling strategies, AUC remains a reliable metric for comparing predictive performance both within and across studies. In general, an AUC greater than 0.8 indicates good discrimination. In the present study, the RF model achieved an AUC of 0.942, substantially outperforming logistic regression and KNN and demonstrating performance comparable to that reported in previous adult HNHC models. Decision curve analysis further confirmed that, within the clinically relevant low threshold probability range of 5–30%, RF–based decision strategies yielded stable net benefit. By contrast, LR performed poorly in this highly imbalanced dataset, suggesting that simple linear assumptions may be insufficient to capture the complex risk patterns underlying ICU cost escalation. Overall, these findings support the use of ensemble learning approaches in critical care management to improve the identification of rare yet clinically and economically important high–resource-utilization events.

Previous studies have shown that certain comorbidities may affect only a single dimension of healthcare utilization (i.e., admission rate, length of stay, or costs), whereas others may influence all three; similarly, demographic factors have also been identified as important predictors of healthcare utilization, although their effects vary depending on the metric used (34). The SHAP-based feature importance ranking in our study provides ICU-specific evidence supporting this perspective. It should be noted that SHAP values quantify each feature’s contribution to the model’s output and do not imply causal relationships between features and healthcare costs. Notably, ICU LOS and MV hours emerged as the two most influential contributors to HNHC classification. This finding is consistent with health economics literature identifying length of stay as a central driver of costs (35); however, our SHAP analysis further refines this understanding by highlighting that prolonged duration alone is not the sole determinant, and that treatment intensity plays a critical role. Prior ICU cost analyses have reported that the average daily cost for patients receiving mechanical ventilation is approximately 1.6 times higher than that for non-ventilated patients (36); the high importance of MV hours in our model—second only to ICU LOS—corroborates this observation. These results suggest that, among HNHC patients, elevated costs are associated not merely from longer ICU stays, but more importantly from sustained exposure to high-intensity respiratory support during hospitalization.

Second, with respect to the effect of age on HNHC risk, our SHAP analysis revealed a more complex nonlinear relationship, extending beyond univariable findings and aligning with established health economics patterns. Although the HNHC group was slightly older on average, age contributed less to the multivariable model than treatment-related factors. As reported in prior studies (37), after adjustment for sex, ICU type, disease severity, length of stay, insurance status, and resuscitation status, older age has been associated with lower total hospitalization costs. This pattern may reflect ethical considerations and generational differences in treatment intensity within ICU decision-making. Clinicians are often more inclined to pursue aggressive, prolonged, and resource-intensive life-sustaining interventions in younger critically ill patients, thereby substantially increasing costs, whereas for patients of very advanced age, care goals may shift earlier toward comfort-focused management or limitations on invasive interventions, partially mitigating the occurrence of extremely high expenditures (38, 39).

Our findings indicate that although HNHC patients were older on average, the SHAP contribution of age in the multivariable model was substantially lower than that of treatment-related variables such as MV hours and transfusion. This is consistent with prior evidence suggesting that (37), in the critical care setting, disease severity and consequent therapeutic interventions are more decisive determinants of resource utilization than biological age per se. These results imply that the emergence of HNHC status is primarily driven by pathophysiology-induced medical actions rather than demographic background alone.

In addition, physiological indicators—including creatinine, platelet count, and CRP—played an important role in the model. Unlike models relying solely on administrative data, the inclusion of laboratory variables enables a more dynamic reflection of organ dysfunction (40). For example, thrombocytopenia often indicates coagulation disorders or sepsis and is commonly associated with increased transfusion requirements and more complex care, thereby indirectly increasing costs (18, 41). Because deterioration in these biological markers typically precedes expensive therapeutic interventions, capturing such changes may contribute to a more comprehensive characterization of patients at high risk of excessive resource consumption.

Beyond population-level feature importance, the individual-level SHAP analyses (Figures 4C–E) provide actionable insights for tiered resource management. For example, in the highest-cost probability case (Figure 4C, *F*(*x*) = 0.995), prolonged mechanical ventilation (62.9 h, SHAP = +0.12), non-postoperative admission (SHAP = +0.09), and transfusion requirement (SHAP = +0.07) were identified as the primary cost-driving factors, while laboratory markers such as platelet count and creatinine exerted minimal influence. Such individualized profiles, when identified through retrospective auditing, could prompt institutional review of whether optimization of MV weaning protocols or transfusion practices was feasible in similar cases. In contrast, low-cost cases were characterized by the absence of organ support and short ICU stays, confirming the appropriateness of standard care pathways. Although this framework is retrospective and not intended for real-time prediction or causal inference, it may inform differentiated cost management protocols and facilitate targeted auditing of high-cost cases to identify potentially modifiable cost drivers for future resource optimization. At the institutional level, the value of this work lies less in algorithmic novelty than in providing ICU-specific, interpretable evidence on the determinants of high-cost utilization in a large multi-specialty cohort. By demonstrating that SHAP-derived contribution patterns persist across the implementation of DRG-based payment reform, our analysis suggests that the identified cost drivers reflect intrinsic features of critical care delivery rather than artifacts of a particular reimbursement context. Such temporally robust, ICU-specific stratification—rarely examined in prior HNHC studies—may assist hospital administrators in identifying cost-driving patterns, support evidence-based budget planning, and inform structured resource-management and reimbursement-refinement protocols under ongoing DRG reform in China.

Several limitations should be acknowledged. First, this was a single-center retrospective study conducted at a large tertiary hospital, and the generalizability of the model to other regions and healthcare settings requires further validation. Second, although key physiological variables (e.g., creatinine, platelet count) were included, composite severity scores such as APACHE II or SOFA were not directly incorporated due to data constraints; however, the ability of ML models to leverage raw continuous variables may have partially mitigated this limitation. Third, MICE imputation was fitted on the full eligible cohort prior to train-test splitting; although our temporally independent validation indicated this did not materially affect model performance, future studies should fit imputation procedures exclusively within the training set as best practice. Fourth, variables were retrospectively extracted from administrative billing data rather than prospectively collected clinical records, and minor discrepancies between billing codes and bedside practice may exist. Fifth, several key predictors—particularly ICU LOS and MV hours—are cumulative variables fully ascertained only at or near ICU discharge; thus, the current model is best characterized as a retrospective risk stratification tool rather than a real-time early warning system. Future research should explore models restricted to variables available within the first 24 h of ICU admission. Finally, only direct medical costs were analyzed; indirect costs and socioeconomic factors (e.g., income or education level) were not available, which may limit a comprehensive assessment of nonclinical contributors to HNHC development.

## 5 Conclusion

This study demonstrates that ICU HNHC patients are characterized by high-technology dependence and acute physiological derangement, with excessive costs associated primarily with organ support therapies—such as MV—and related consumables rather than with demographic factors alone. By integrating treatment duration, intervention intensity, and laboratory indicators, a random forest–based ML model effectively addressed class imbalance and achieved accurate classification of high-cost risk. Importantly, our findings indicate that the mechanisms underlying resource consumption among critically ill patients remain temporally stable and do not exhibit clinically meaningful shifts in response to DRG-based payment reform. This stability underscores the robustness of classification models built on core clinical features and supports their use as reliable tools to facilitate structured retrospective risk stratification and more precise resource allocation in critical care setting.

## Data Availability

The original contributions presented in the study are included in the article/Supplementary material, further inquiries can be directed to the corresponding author.
