# Supplementary material for: Risk stratification and determinant identification of high-need, high-cost ICU patients using machine learning: a large-scale retrospective study from a multi-specialty ICU in a tertiary hospital
Source: Front Public Health. 2026 May 29;14:1783334. doi: 10.3389/fpubh.2026.1783334 (PMC13259673; doi:10.3389/fpubh.2026.1783334)
Supplement: Supplementary file 1 [file Supplementary_file_1.DOCX]

**Table S1. Annual distribution of ICU admissions, HNHC patient proportions, and cost thresholds (95th percentile) stratified by DRG implementation period.**

| **Year** | **DRG Period** | **Total ICU Admissions** | **HNHC Patients (n)** | **HNHC Proportion (%)** | **95th Percentile Threshold (CNY)** | **HNHC Cost Range (CNY)** | **Cross-year Admissions (n)** |
| --- | --- | --- | --- | --- | --- | --- | --- |
| 2018 | Pre-DRG | 7169 | 359 | 5 | 138344.68 | 138352–346033 | 73 |
| 2019 | Pre-DRG | 7015 | 351 | 5 | 144606.07 | 144744–318959 | 65 |
| 2020 | Pre-DRG | 5417 | 271 | 5 | 151818.23 | 151852–336667 | 60 |
| 2021 | Post-DRG | 7550 | 378 | 5 | 148853.46 | 148854–346959 | 84 |
| 2022 | Post-DRG | 5488 | 275 | 5 | 158548.50 | 158718–337677 | 56 |
| 2023 | Post-DRG | 8756 | 438 | 5 | 148741.50 | 148763–330465 | 87 |
| 2024 | Post-DRG | 9661 | 484 | 5 | 147340.74 | 147341–335985 | 0 |
| **Overall** | 2018–2024 | 51056 | 2556 | 5 | 148237.21 | 138352–346959 | 425 |

**Table S2. Detailed algorithm principles, hyperparameter search ranges, and optimization results for the six machine learning models.**

| **Model** | **Algorithm Principle** | **Rationale for Inclusion** | **Key Hyperparameters**  **(Search Range)** | **Optimized Values** | **Optimal Threshold** |
| --- | --- | --- | --- | --- | --- |
| **Random Forest (RF)** | Bagging ensemble of decision trees; each tree is trained on a bootstrap sample with a random subset of features (mtry) at each split; final prediction by majority vote | Robust to mixed feature types (continuous + categorical); reduces variance via bootstrapping and feature randomization; handles nonlinear relationships well | n_estimators: 100–300 max_depth: 5–20 min_samples_split: 5–30 min_samples_leaf: 2–15 max_features: sqrt / log2 | n_estimators = 177 max_depth = 20 min_samples_split = 6 min_samples_leaf = 2 max_features = sqrt | 0.60 |
| **Extra Trees (ET)** | Similar to RF but uses fully random split thresholds instead of optimal splits; further increases randomization to reduce variance | Additional variance reduction compared to RF; suitable for large-scale datasets; provides a complementary bagging-based comparison | n_estimators: 100–300 max_depth: 5–20 min_samples_split: 5–30 min_samples_leaf: 2–15 max_features: sqrt / log2 | n_estimators = 100 max_depth = 20 min_samples_split = 5 min_samples_leaf = 2 max_features = sqrt | 0.73 |
| **XGBoost** | Gradient boosting framework; sequentially adds decision trees to minimize a regularized loss function with L1 (α) and L2 (λ) penalty terms | Strong performance on imbalanced data; built-in regularization prevents overfitting; handles complex nonlinear feature interactions | n_estimators: 100–300 max_depth: 3–10 learning_rate: 0.01–0.3 subsample: 0.6–1.0 colsample_bytree: 0.6–1.0 reg_alpha: 0.01–10 reg_lambda: 0.01–10 | n_estimators = 300 max_depth = 10 learning_rate = 0.30 subsample = 0.6 colsample_bytree = 1.0 reg_alpha = 0.01 reg_lambda = 0.01 | 0.10 |
| **LightGBM** | Histogram-based gradient boosting with leaf-wise tree growth strategy; uses Gradient-based One-Side Sampling (GOSS) for efficiency | Significantly faster training on large datasets (50,000+ samples); built-in L1/L2 regularization; efficient handling of high-cardinality categorical features | n_estimators: 100–300 max_depth: 3–15 learning_rate: 0.01–0.3 num_leaves: 20–100 min_child_samples: 10–50 reg_alpha: 0.01–10 reg_lambda: 0.01–10 | n_estimators = 300 max_depth = 15 learning_rate = 0.30 num_leaves = 100 min_child_samples = 10 reg_alpha = 0.01 reg_lambda = 10.0 | 0.21 |
| **K-Nearest Neighbors (KNN)** | Instance-based non-parametric method; classifies samples by majority vote of k nearest neighbors based on distance metrics | Non-parametric baseline without model assumptions; evaluates whether distance-based simple methods are competitive for this task | n_neighbors: 5–15 weights: distance metric: euclidean / manhattan | n_neighbors = 5 weights = distance metric = manhattan | 0.10 |
| **Logistic Regression (LR)** | Linear classification model: P(Y=1\|X) = 1/(1+exp(-(β₀+βᵀX))) with L2 regularization penalty | Interpretable baseline model; establishes a linear benchmark against which complex models can be compared; widely used in clinical prediction | C: 0.001–100 penalty: L2 solver: lbfgs / saga | C = 15.38 penalty = L2 solver = lbfgs | 0.85 |

**Note:** All hyperparameters were tuned via Bayesian Optimization with 5-fold cross-validation (F1-score as objective, 50 iterations per model). Classification thresholds were optimized on the training set by maximizing F1-score. Implementation: Python 3.9.13, scikit-learn, XGBoost, LightGBM, imbalanced-learn.

**Table S3. Comparison of hospitalization cost components between HNHC and non-HNHC ICU patients**

| **Variables** | **Overall(N=51056)** | **non-HNHC (n=48500)** | **Non-HNHC (n=2556)** | **P-value** |
| --- | --- | --- | --- | --- |
| **Hospitalization costs (10,000 CNY)** |  |  |  |  |
| Total hospitalization cost, median (IQR) | 7.79 (4.03-11.86) | 7.30 (3.88-11.26) | 22.58 (19.41-27.41) | <0.001 |
| ICU cost, median (IQR) | 4.23 (2.27-7.12) | 4.04 (2.18-6.55) | 18.81 (16.53-22.91) | <0.001 |
| Drug cost, median (IQR) | 0.31 (0.16-0.72) | 0.29 (0.16-0.65) | 2.39 (0.93-5.10) | <0.001 |
| Material cost, median (IQR) | 2.12 (0.50-4.22) | 1.96 (0.45-3.90) | 10.60 (6.09-13.49) | <0.001 |
| Laboratory test cost, median (IQR) | 0.33 (0.20-0.51) | 0.32 (0.20-0.48) | 1.24 (0.52-2.18) | <0.001 |
| Imaging examination cost, median (IQR) | 0.28 (0.14-0.51) | 0.26 (0.14-0.48) | 0.98 (0.51-1.63) | <0.001 |
| Surgery cost, median (IQR) | 0.03 (0.00-0.49) | 0.03 (0.00-0.49) | 0.00 (0.00-0.44) | <0.001 |
| Treatment cost, median (IQR) | 0.15 (0.06-0.60) | 0.14 (0.06-0.58) | 1.02 (0.41-1.64) | <0.001 |
| Nursing cost, median (IQR) | 0.05 (0.02-0.10) | 0.04 (0.02-0.09) | 0.19 (0.06-0.53) | <0.001 |

**Table S4. Statistical comparison of SHAP value distributions for key predictors between the pre-DRG and post-DRG periods.**

| **Feature** | **Pre-DRG Mean SHAP** | **Pre-DRG SD** | **Post-DRG Mean SHAP** | **Post-DRG SD** | **P-value** |
| --- | --- | --- | --- | --- | --- |
| Age | -0.0179 | 0.0211 | -0.0160 | 0.0234 | <0.0001 |
| ICU LOS | -0.0918 | 0.0962 | -0.0969 | 0.0951 | 0.0091 |
| Secondary Dx | -0.0378 | 0.0282 | -0.0388 | 0.0311 | <0.0001 |
| MV Hours | -0.0722 | 0.0865 | -0.0646 | 0.0874 | <0.0001 |
| CRRT Hours | -0.0099 | 0.0149 | -0.0093 | 0.0186 | <0.0001 |
| ECMO Hours | -0.0065 | 0.0165 | -0.0071 | 0.0125 | <0.0001 |
| Creatinine | -0.0102 | 0.0101 | -0.0109 | 0.0118 | 0.4783 |
| Platelet | -0.0119 | 0.0159 | -0.0142 | 0.0190 | <0.0001 |
| CRP | -0.0124 | 0.0159 | -0.0105 | 0.0154 | <0.0001 |
| Lymphocyte | -0.0103 | 0.0152 | -0.0093 | 0.0162 | <0.0001 |
| Sex | -0.0047 | 0.0104 | -0.0051 | 0.0124 | 0.1678 |
| Postop | -0.0143 | 0.0294 | -0.0141 | 0.0323 | 0.3702 |
| Intubation | -0.0095 | 0.0106 | -0.0055 | 0.0180 | <0.0001 |
| Transfusion | -0.0212 | 0.0397 | -0.0173 | 0.0396 | <0.0001 |
| DRG | -0.0037 | 0.0066 | -0.0041 | 0.0052 | 0.0467 |
| Payer | -0.0023 | 0.0059 | -0.0016 | 0.0064 | <0.0001 |
| Admit Source | -0.0096 | 0.0181 | -0.0103 | 0.0197 | 0.044 |
| ICU Type | -0.0256 | 0.0422 | -0.0240 | 0.0444 | 0.0024 |
| Primary Dx | -0.0086 | 0.0393 | -0.0083 | 0.0433 | <0.0001 |

**Table S5.Stratified performance of the Random Forest model before and after DRG payment reform (with 95% bootstrap confidence intervals).**

| **Group** | **AUC** | **PR-AUC** | **Brier Score** | **F1** | **Sensitivity** | **Specificity** |
| --- | --- | --- | --- | --- | --- | --- |
| Pre-DRG (2018-2020) | 0.947 (0.932-0.961) | 0.624 (0.546-0.697) | 0.039 (0.0355-0.042) | 0.594 (0.526-0.655) | 0.589 (0.516-0.665) | 0.980 (0.975-0.984) |
| Post-DRG (2021-2024) | 0.938 (0.923-0.950) | 0.612 (0.549-0.667) | 0.045 (0.0421-0.047) | 0.580 (0.533-0.623) | 0.609 (0.555-0.660) | 0.974 (0.970-0.977) |

**Note：**ΔAUC = 0.010; 95% bootstrap CI of the difference: −0.011 to 0.030; the CI included zero, indicating no statistically significant difference.

**Table S6. Sensitivity analysis comparing the performance of different class imbalance handling strategies on the independent testing set.**

| **Strategy** | **AUC** | **AUC (95% CI)** | **PR-AUC** | **F1** | **F1 (95% CI)** | **Sensitivity** | **Specificity** | **Precision** | **MCC** | **Threshold** |
| --- | --- | --- | --- | --- | --- | --- | --- | --- | --- | --- |
| Cost-sensitive | 0.9447 | 0.9448 (0.9342-0.9544) | 0.6488 | 0.5877 | 0.5874 (0.5469-0.6261) | 0.4951 | 0.9900 | 0.7229 | 0.5814 | 0.55 |
| Random Oversampling | 0.9438 | 0.9440 (0.9333-0.9534) | 0.6403 | 0.5304 | 0.5307 (0.4881-0.5726) | 0.3836 | 0.9967 | 0.8596 | 0.5612 | 0.70 |
| SMOTE + Tomek | 0.9421 | 0.9423 (0.9316-0.9522) | 0.6103 | 0.5875 | 0.5874 (0.5529-0.6219) | 0.6145 | 0.9748 | 0.5627 | 0.5653 | 0.59 |
| SMOTE | 0.9417 | 0.9418 (0.9309-0.9518) | 0.6138 | 0.5856 | 0.5858 (0.5504-0.6203) | 0.6027 | 0.976 | 0.5693 | 0.5633 | 0.60 |
| No Resampling | 0.9409 | 0.9410 (0.9296-0.9517) | 0.6723 | 0.6045 | 0.6047 (0.5684-0.6403) | 0.6086 | 0.9787 | 0.6004 | 0.5835 | 0.26 |
| ADASYN | 0.9406 | 0.9408 (0.9299-0.9508) | 0.6076 | 0.5898 | 0.5896 (0.5556-0.6219) | 0.6106 | 0.9758 | 0.5704 | 0.5678 | 0.60 |

**
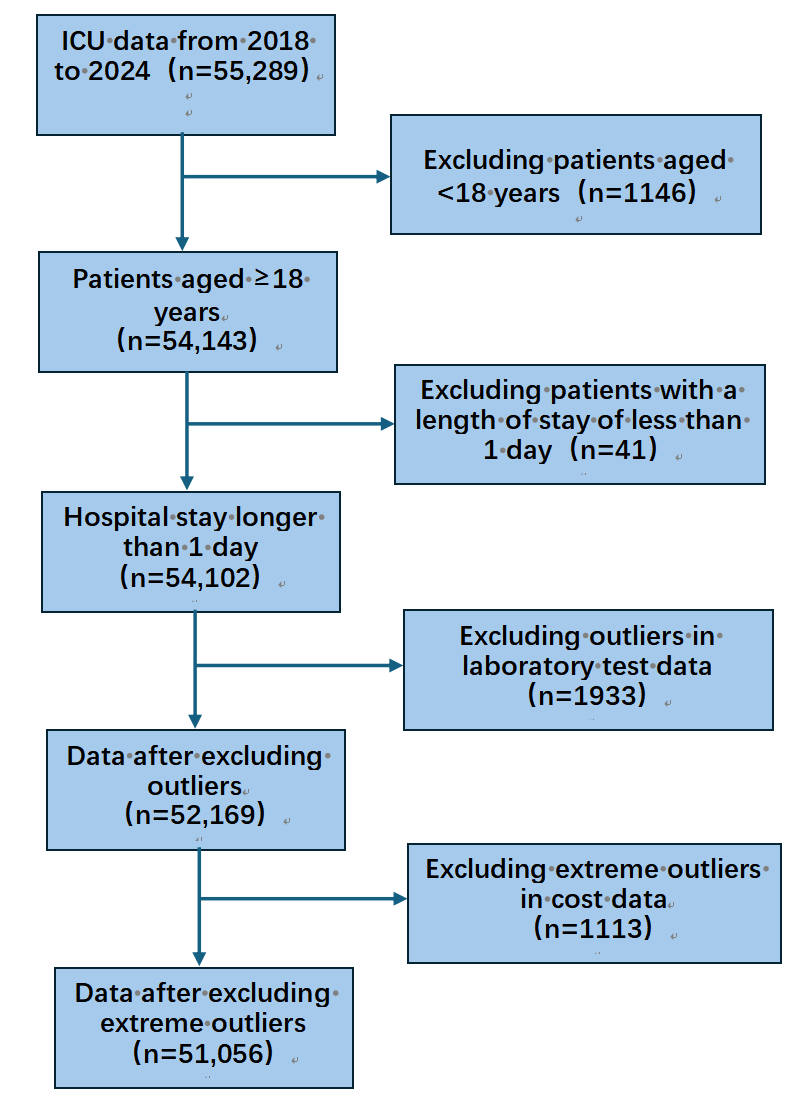
Figure S1. Flowchart of ICU patient selection and data cleaning process.**

**
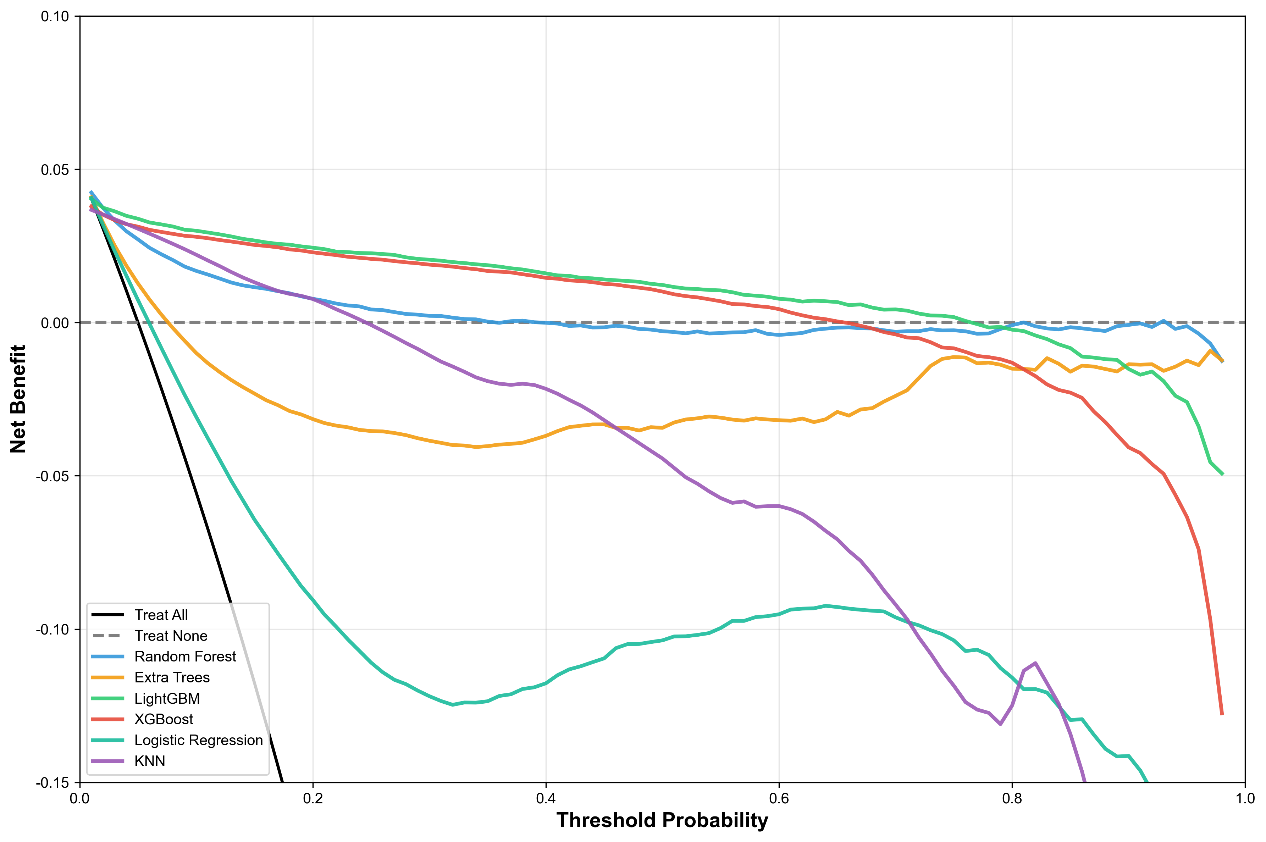
Figure S2. Decision curve analysis of machine learning models in the testing set**

**
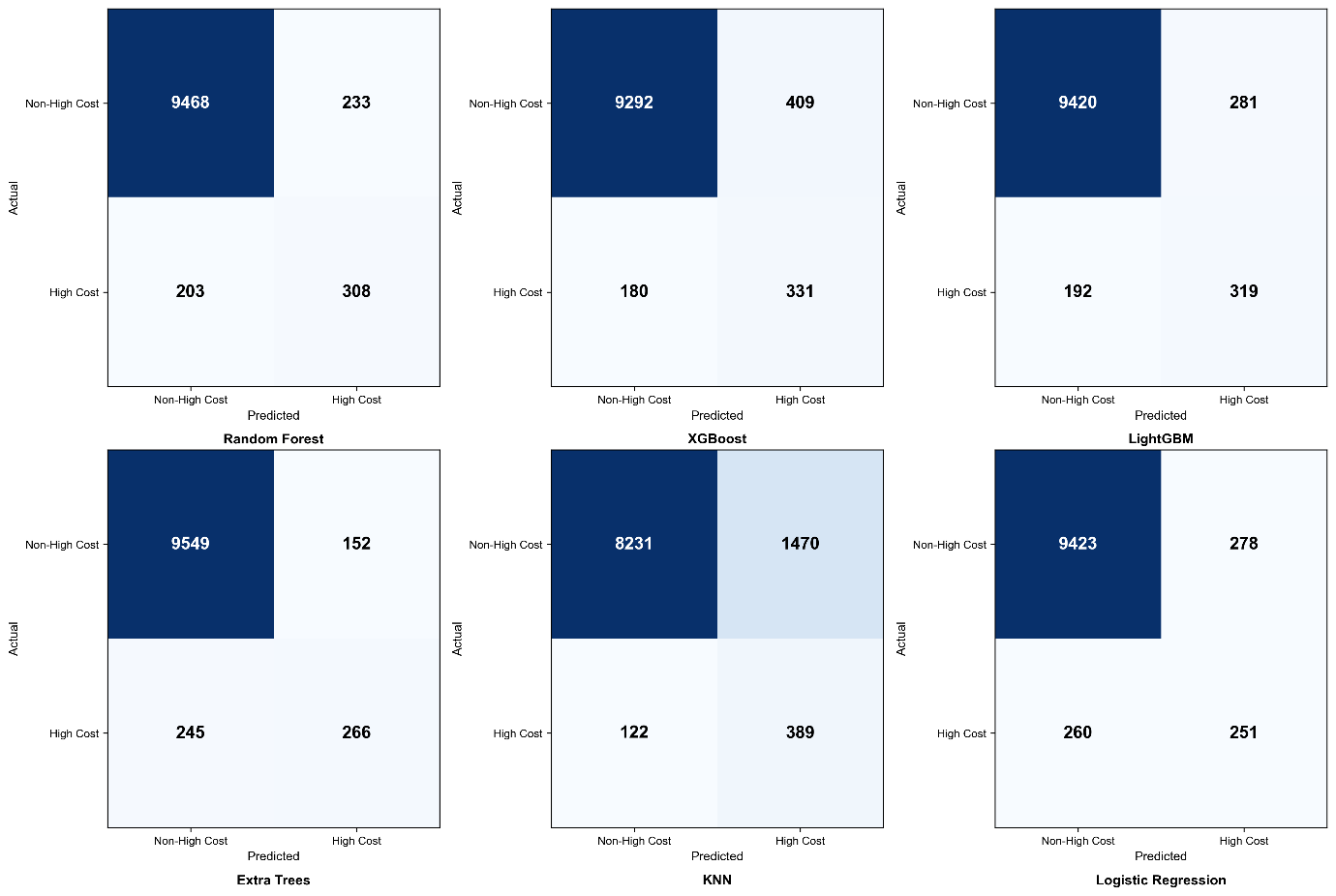
Figure S3. Confusion matrix performance of multiple machine learning models in predicting high-need, high-cost ICU patients**

**
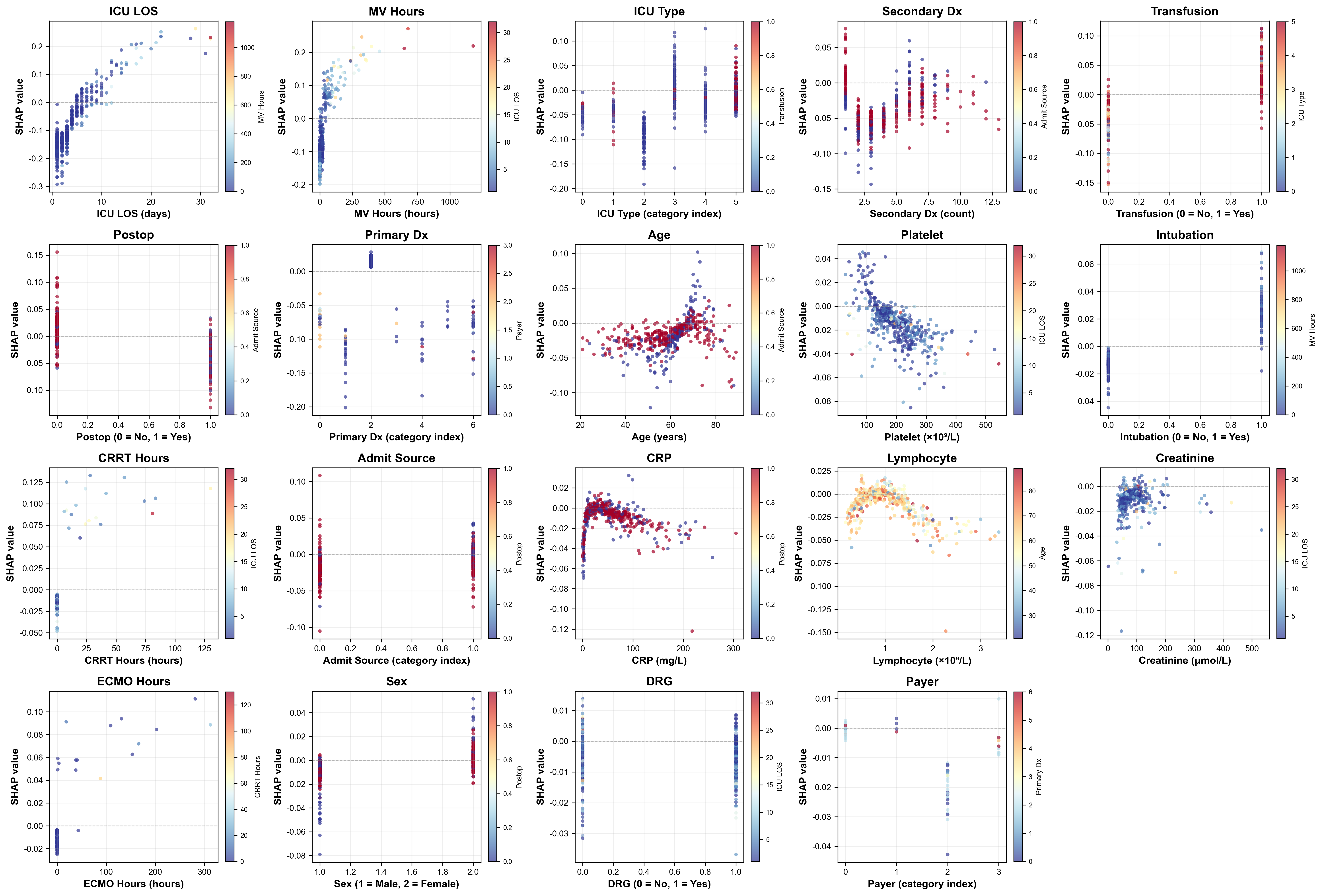
Figure S4. SHAP scatter plots showing the impact and direction of clinical features on the prediction of high-need, high-cost ICU patients**

**
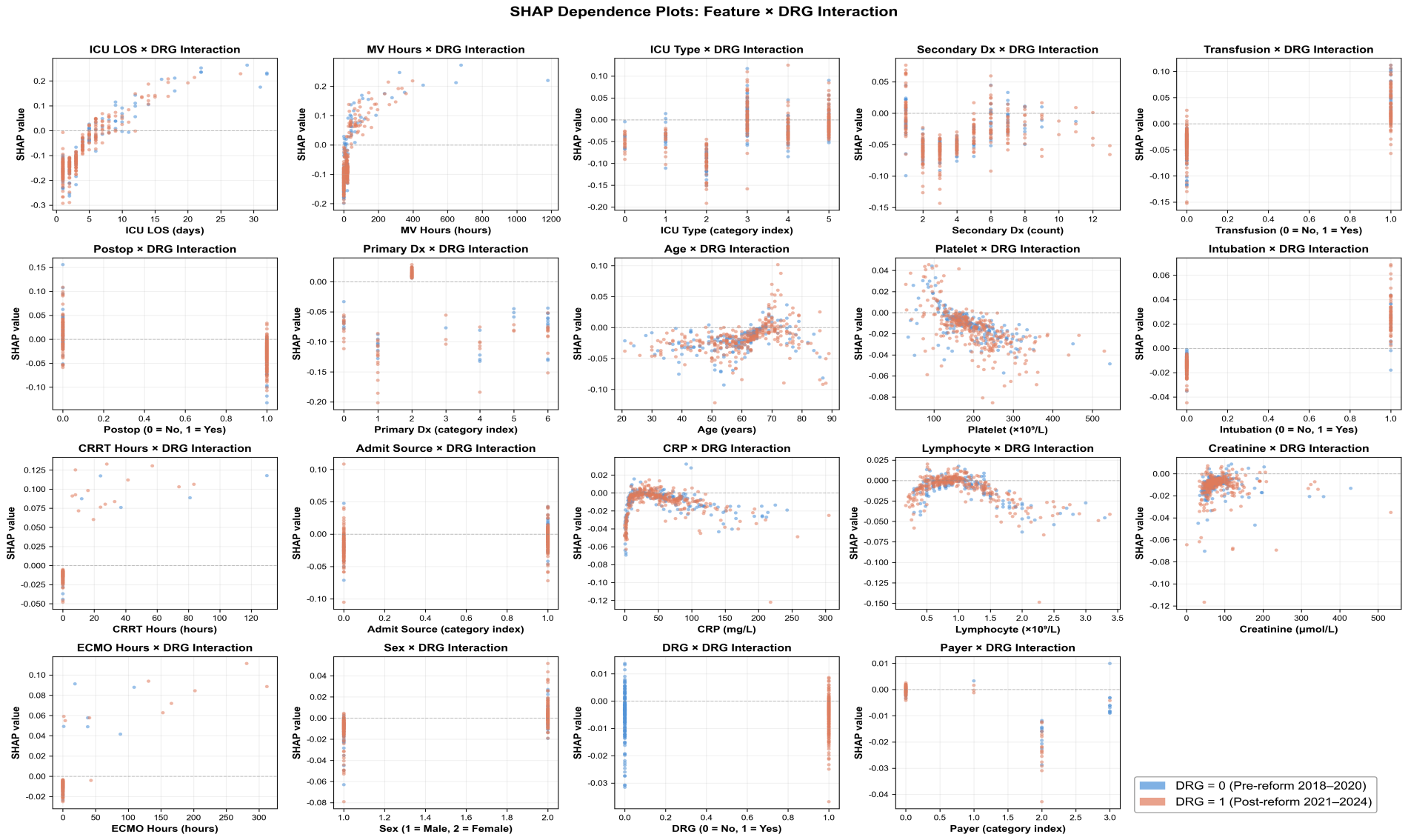
Figure S5. SHAP dependence plots illustrating interactions between clinical features and DRG reform status in predicting high-need, high-cost ICU patients**

**Figure S6. Stratified performance of the Random Forest model before and after DRG payment reform. (A) ROC curves; (B) Precision-Recall curves; (C) Calibration plot for the pre-DRG period (2018–2020); (D) Calibration plot for the post-DRG period (2021–2024)**

**
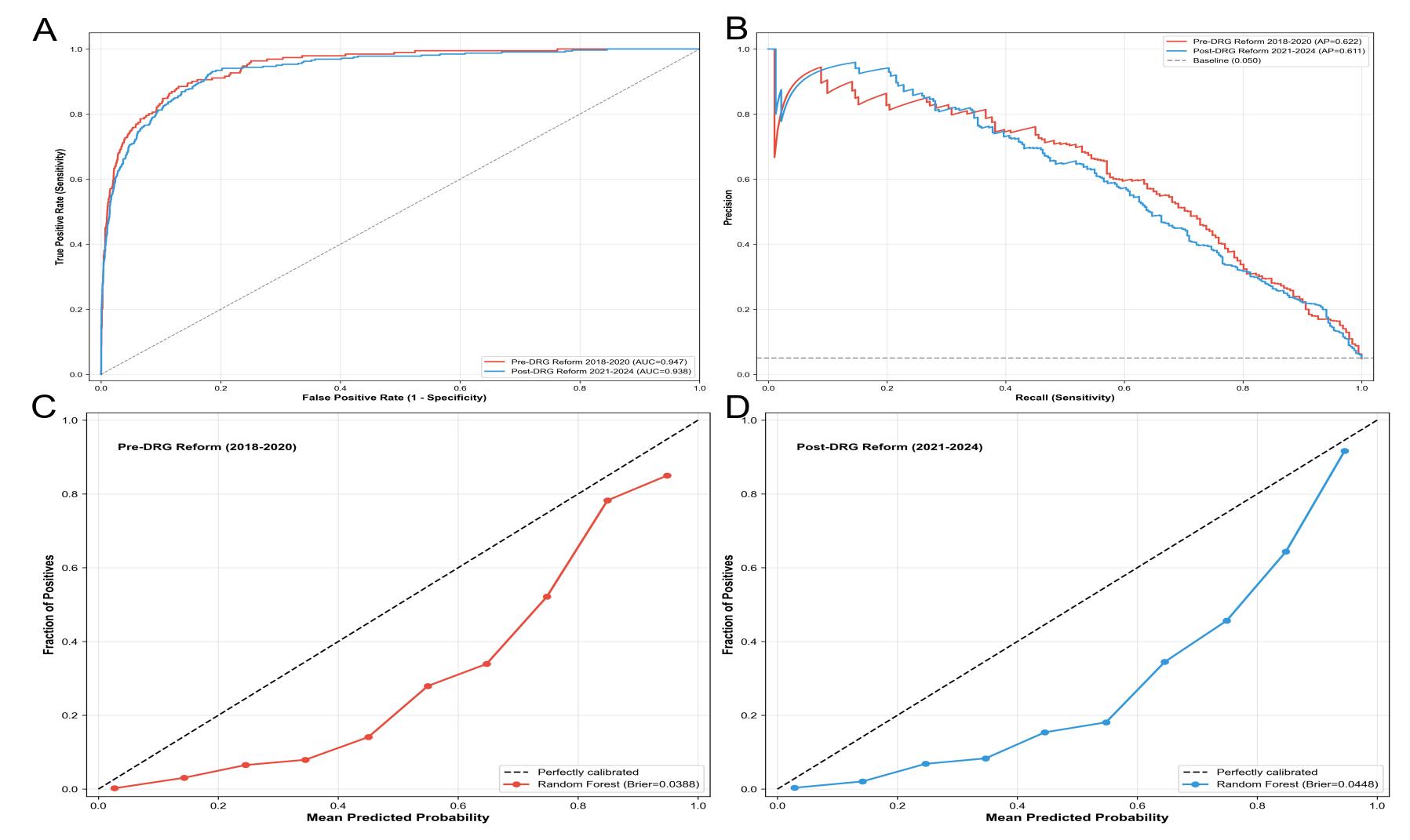
**
